# 

**Published:** 1912-07

**Authors:** 


### INDEX FOR VOL. LXVII

### AUGUST, 1911, to JULY, 1912

| Page | |
|---|---|
| 1— 60 | August |
| 61—120 | September |
| 121—180 | October |
| 181—242 | November |
| 243—304 | December |
| 305—364 | January |
| 365—420 | February |
| 421—476 | March |
| 477—538 | April |
| 539—600 | May |
| 601—662 | June |
| 663—716 | July |

CONTRIBUTORS:—

ASHCRAFT, E. H., Coudersport, Pa., Austin Flood, page 196, Nov., 1911.

BARTOW, Bernard, Buffalo, (and Wm. Ward Plummer), Use of Intra-articular Silk Ligaments for Fixation of Loose Joints in Residual Paralysis of Anterior Poliomyelitis, page 310, Jan., 1912.

Bebee, Edwin L., Buffalo, Vicious Union of Shaft of Femur, page 253, Dec., 1911.

Bethune, Charles W., Buffalo, Treatment of Acute Gonorrhoea, page 121, Oct., 1911; Treatment of Subacute Gonorrhoea, 185, Nov., 1911; Treatment of Chronic Gonorrhoea, 256, Dec., 1911.

Brust, H. O., Syracuse. See Wallace.

COTT, Chester C., Buffalo, Suppurative Labyrinthitis, 672, July, 1912.

Cott, George F., Buffalo, Latent Sinusitis, page 1, Aug. 1911; Review of 100 Radicals, page 243, Dec., 1911.

Cowper, H. M., Buffalo, Penetrating Wound of Orbit with Dislodgment of Vomer, page 336, Jan., 1912.

Crego, Floyd S., Buffalo, Meningitis, page 421, March, 1912.

Crothers, T. D., Hartford, Conn., Some Statistic Studies of the Cure of Inebriety, page 247, Dec., 1911.

FELL, George E., Buffalo, Sanitation of Cities of the Niagara Frontier, page 486, Apr., 1912.

Foy, George, Dublin, Ireland. Unusual Case of Injury of Thorax, page 604, June, 1912.

Fronczak, Francis E., Buffalo, The Law as it Relates to Physicians and the Buffalo Health Department, page 477, Apr., 1912; page 563, May, 1912; The Duties of Physicians in Regard to Insanity, page 604, June, 1912.

GARDNER, James A., Buffalo, Use of Silver Iodide in Urethritis and Cystitis, page 321, Jan., 1912; Inflammation of the Veru Montanum and Posterior Urethra with Presentation of an Instrument, page 609, June, 1912.

Garlock, W. S., Little Falls, N. Y., Serum Therapy, page 556, May, 1812.

Goldsborough, Francis C., Buffalo, Labor in Moderately Contracted Pelves, page 126, Oct., 1191.

Gould, George M., Ithaca, Heterophoria Superstition, page 79, Sept., 1911; Denutrition, Migraine, Esophoria, Fading Image, etc., due to Eye Strain, page 324, Jan., 1912.

Griswold, V. M., Fredonia, N. Y., Vincent's Angina, page 431, March, 1912.

HATCH, Edith R., Buffalo, Acute Pyelitis in Infancy, page 663, June, 1912.

Howell, Stephen Y., Buffalo, Dysmenorrhoea, page 543, May, 1912.

KNOLL, John G. W., Buffalo, Technic, page 446, March, 1912; Report of Cases Treated with Gonococcus Vaccine, page 543, May, 1912.

LEE, John M., Rochester, Retroperitioneal Tumors and Intraligamentary Pregnancy, page 539, May, 1912.

Lewis, F. Drake, Buffalo, Conservative Ophthalmology, page 61, Sept., 1911.
Litchtenberg, H. F., Buffalo, Polarimetry as Applied, to Urinalysis page 190, Nov., 1911.
Little, S. W., Rochester, A New Departure in the Medical Expert Matter, page 552, May, 1912.
Long, Eli H., Buffalo, Timing of Heart Murmurs, page 375, Feb., 1912.

MAYO, William J., Rochester, Minnesota, Carcinomata of Gastor-intestinal Tract, page 305, Jan., 1912.
McGuire, Edgar R., Buffalo, Perforation in Doudenal Ulcer, page 613, June, 1912.
McMichael, George H., Buffalo, Buffalo Hospital for Alcoholics, page 278, Dec., 1911.
Miller, Joseph L., Chicago, Normal and Pathology of the Hypophysis, page 365, Feb., 1912.

NOEHREN, Alfred H., Buffalo, Traumatic Finger Amputations, page 605, June, 1912.

PARKER, Esther E., Ithaca, Facts Concerning Physical Condition of Women During College Life, page 666, July, 1912.
Plummer, William Ward, Buffalo, See Bartow.
Potter, W. W., Buffalo, Three Years with the Army of the Potomac, 18, 69, 132, 199, 261, 326, 377, 436, 500, 552, 625, 678, Aug., 1911—July 1912.
Putnam, James Wright, Buffalo, (and Sharp), Case of Myoclonus Associated with Unilateral Epileptoid Convulsions, page 181, Nov., 1911.

QUINTON, W. W., Buffalo, Treatment of Syphilis, page 14, August, 1911.

SHARP, Edward A., Buffalo, See Putnam.
Smith, Chauncey P., Buffalo, Results from Use of Dowd's Phosphatometer and Tonic, page 497, Apr., 1912.
Snow, Sargent F., Syracuse, Colds, Causes and Treatment, page 9, Aug., 1911.
Swain, Frank S., Corning, N. Y., Typhoid Epidemic in Corning, page 622, June, 1912.

WALLACE, W. L., and H. O. Burst, Syracuse, Case of Laceration of Trachea, page 130, Oct. 1911.
Whipple, E. G., Rochester, Public Health Association, page 504, Apr., 1912.

OBITUARIES:—

Abbott, George, August, 1911. .... 36
Adam, J. N., Mar., 1912 ........ 471
Babcock, Oliver H., Aug., 1911 ..112
Baldwin, Mary, Mar., 1912 .......469
Barton, Clara, May, 1912 .......587
Barcroft, Wm. H., May, 1912 ...585
Belding, Dexter R., July, 1912 ....710
Bleakney, Samuel M., Mar., 1912, 470
Brown, Chas. W. M., Dec., 1911, 291
Casey, Philip, Dec., 1911 ........291
Clark, Geo., Oct., 1911 ..........171
Clowe, Chas. F., May, 1912 ..... 654
Collins, Newton M., July, 1912 ..710
Chessman, Wm. S., May, 1912 ..654
Cronyn, Mrs. Elizabeth Ann Renfew Willoughly, Feb., 1912 ....413
Davis, John D., May, 1912 ........587
Dorr, Samuel H., April, 1912 ....528
DuBois, Hiram Gettman, Nov., 1911 ............................232
Dwight, Thomas, Nov., 1911 .....232
Eggleston, H. Warner, May, 1912, 585
Evans, O. E., Jan., 1912 ........353
Ferguson, Alex. Hugh, Dec., 1911, 291
Fish, Everett W., April, 1912 ...531
Freer, Paul C., May, 1912 ....... 585
Frederick, Carlton C., Aug., 1911, 35
Foster, Frank, Sept., 1911 ..... 112
Green, Chas. O., Sept., 1911 ... 112
Greenleaf, Chas. Ravencroft, Nov., 1911 ............................232
Halbert, John Sanford, Aug., 1911, 38
Hall, Oscar H., May, 1912 .......587
Herbst, Henry Herbert, Nov., 1911 232
Hewitt, James H., April, 1912 ...530
Hix, Ira A., May, 1912 ..........588
Horton, Eugene B. May, 1912 ..654
Horton, Levi E., May, 1912 ......654
Hubbell, Alvin A., Sept., 1911 .... 96
Nov., 1911 .................233
James, Ephraim P., May, 1912 ..585
Johnson, Walter W., Mar., 1912..468
Jones, John N., Nov., 1911 ......232
Klinington, Samuel S., Nov., 1911, 233
King, Leroy Wendell, July, 1912, 710
Kingsley, Willey J. P., Mar., 1912, 468
Kuhles, Philip, Mar., 1912 .......468
Lapp, Henry, April, 1912 .......530
Lewis, J. A., Sept., 1911 ........112
Little, David, April, 1912 .......529
Lord, Lister, Mar., 1912 .........469
McCaw, Will. Dec., 1911 .......292
McGill, Wm. D, Dec., 1911 ......292

Maxson, Edwin R., April, 1912 ..529
Meary, Edward, Jan., 1912 ......354
Miller, John H., Dec., 1911 ......291
Moody, Horace M., Mar., 1912 ...470
Musser, John H., May, 1912 .....584
Nelson, Lewis, May, 1912 ......585
Nichols, Chas. W., May, 1912 ....654
O'Brien, Pierce J., July, 1912 ....710
Pearsons, Daniel K., May, 1912 ..654
Phelps, Wm. C., Sept., 1911 ....111
Nov., 1911 ..................233
Potter, William Warren, Aug., 1911 ............................ 35
Rasbach, James I., May, 1912 ....587
Reynolds, Geo. H., May, 1912 ..654
Reynolds, Geo. Wm., Nov., 1911, 232
Rice, Leroy George, Jan., 1912, 353
Richards, Ira Dwight, Sept., 1911, 112
Rinkle, Lafayette, Jan., 1912 ....353
Rolph, Rollin Thomas, July, 1912, 710
Ross, Wm. Frederick James, Jan., 1912 .......................... 354
Rose, Lewis Wheeler, Oct., 1911, 171
Salinger, Julius L., May, 1912, ..568
Sayles, George W., Sept., 1911 ..112
Schroeter, Ludwig, Apr., 1912 ....350
Seitz, Frank B., Aug., 1911 ...... 35
Shumway, John P., May, 1912 ..587
Smith, Charles, May, 1912 .......585
Smith, James S., March, 1912 ..467
Smith, T. Guilford, March, 1912 ..471
Snyder, Fairfield, Feb., 1912 ....413
Sternberg, James H., May, 1912 .587
Straight, A. B., Feb., 1912 ......413
Strassenburg, George, Feb., 1912, 413
Sutton, Frank L., March, 1912 ..470
Taylor, John Yeatman, Dec., 1911, 291
Thomas, Arthur W., Apr., 1912 .529
Tremaine, Annie W., Apr., 1912, 530
Trowbridge, Frederick G., Nov., 1911 .......................... 233
Trowbridge, Lawrence, Apr., 1912, 531
Tryon, James R., Apr., 1912 ......531
Vadeboncouer, Antoine F., Dec., 1911 .......................... 291
Walker, Henry O., May, 1912 ..586
Wasdin, Eugene, Dec., 1911 ......292
Welch, Bennett T., Dec., 1911 ...291
Wells, Willis M., May, 1912 ......587
Wenz, Julius, June, 1912 ........654
Wheeler, William A., March, 1912, 468
Whitford, William J., June, 1912, 654
Wilbur, Leonidas Franklin, Oct., 1911 ........................... 171
Wilson, Benjamin, Nov., 1911 ....232
Williams, Isaac R., Feb., 1912 ....413
Wood, Edwin L., July, 1912 ......710
Wyman, Walter, Dec., 1912 ......292

**BOOK REVIEWS:—**

AARON, Charles D., Diseases of the Stomach, with Special Reference to Treatment, Nov., 1911.
Aitken, John, Principles of Anatomy and Physiology, Sept., 1911.
Aitken, Charles A., Hospital Management, Sept., 1911.
Aitkens, Charlotte A., Home Nurse's, a Handbook of Practical Training, page 713, July, 1912.
Andrews, James M., Andrew's and Boston's Medical Diagnosis, page 301, Dec., 1911.
Andrews, James M., Practice of Medicine, page 363, Jan., 1912.
American Ethnology, Twenth-Seventh Annual Report of the Bureau, for year 1905-6, page 474, March, 1912.
American Larynogolozic, Rhinologic, Otologic Society, Transaction of the 17th Annual Meeting of the, June, 1911, page 595, May, 1912.
American Laryngological Association, Transactions of the 33rd Annual Meeting of the,page 534, April, 1912.
American Association of Genito-Urinary Surgeons, Transactions of the, page 535, April, 1912.

BAILEY, Frederick Randolph, Text Book of Embryology, page 361, Jan., 1912.
Bastian, H. Carlton, The Origin of Lif Dec., 1911.
Bechamp, A., The Blood and its Third Anatomical Element, page 415, Feb., 1912.
Bidwell, Leonard A., Minor Surgery, page 414, Feb., 1912.
Blair, Thomas, Blair's Pocket Therapeutica, page 532, April, 1912
Blakiston's, P. Son & Co., Physician's Visiting List, page 361, Jan., 1912.
Bradford, E. H., Orthopedic Surgery, page 420, Feb., 1912.
Brickner, Walter M., Surgical Suggestions, Aug., 1911.
Bryant, Joseph D., American Practice of Surgery, Aug., 1911.
Buchanan, Robert E., Veterinary Bacteriology, page 358, Jan., 1912.
Bull, W. Sheldon, Money in Goats, page 597, May., 1912.
Bulkley, L. Duncan, Compendium of Diseases of the Skin, page 657, June, 1912.
Butler, Reeve Glentworth, The Diagnostica of Internal Medicine, page 419, Feb., 1912.

CABOT, Richard C., Case Histories in Medicine, page 415, Feb., 1912.
Cabot, Richard C., Differential Diagnosis, page 656, June, 1912.
Cattell, Henry W., Lippincott's New Medical Dictionary, Dec., 1911.
Chapin, Henry Dwight, Diseases of Infants and Children, page 357, Jan., 1912.
Church-Peterson, Nervous and Mental Diseases, page 531, April, 1912.
Citron, Julius, Immunity, page 659, June, 1912.
Commissioner of Education, Report of the, page 657, June, 1912.
Collins, Joseph, The Way with the Nerves, page 364, Jan., 1912.
Cornell, Walter S., Health and Medical Inspection of School Children, page 532, Apr., 1912.
Coplin, W. M. Late, Manual of Pathology, page 299, Dec., 1911.
Collected Papers, Staff of St. Mary's Hospital, page 359, Jan., 1912.

DA COSTA, John C., Principles and Practice of Physical Diagnosis, page 533, April, 1912.
Darmstadt, Merck's Annual Report of Recent Advances in Pharmaceutical Chemistry and Therapeutica, page 596, May, 1912.
Davis, Edward, Operative Obstretica, page 594, May., 1912.
Davis, Gwilym, The Principles and Practice of Bandaging, Dec., 1911.
Delafield, Francis, Text Book of Pathology, page 362, Jan., 1912.
Denslow, L. N., Surgical Treatment of Locomotor Ataxia, page 659, June, 1912.
Ditman, Norman E., Home Hygiene and Prevention of Disease, page 593, May, 1912.
Lee, Frederic S., Scientific Features of Modern Medicine, page 414, Feb., 1912.

EINHORN, Max, Diseases of the Stomach, page 359, Jan., 1912.
Ellis, Havenlock, Sex in Relation to Society, page 300, Dec., 1911.
Ely, Leonard, Recent Progress in Orthopedic Surgery, page 471, Mar., 1912.
Ely, Leonard W., Joint Tuberculosis, Sept., 1911.

FITCH, William, New Pocket Medical Formulary with an Appendix, page 658, June 1912.

GOULD, George M., A Pocket Medical Dictionary, Oct., 1911.
Green, T. H., A Manual of Pathology and Morbid Anatomy, Dec., 1911.

HALLIBURTON W. D., Handbook of Physiology, page 474, Mar., 1912.
Hare-Appelman, Progressive Medicine, page 533, April, 1912., page 712, July, 1912.
Harrington, Charles, A Manuel of Practical Hygiene, Nov., 1911.
Head, Gustave P., The Practical Medicine Series, Oct., 1911, Aug., 1911.
Head, G. P., The Practical Medicine Series, Nov., 1911, page 713, July., 1912.
Hill, Hon. Henry W., The Champlain Tercentenary, page 473, Mar., 1912.
Hirschberg-Johnson, Treatment of Short Sight, page 655, June, 1912.
Honan, James H., Honan's Handbook to Medicial Europe, page 531, April, 1912.
Holland, James W., Medical Chemistry and Toxicology, page 362, Jan., 1912.
Howell, William H., Text Book of Physiology, page 356, Jan., 1912.
Handbook of the Anglo-American Medical Association of Berlin, page 658, June, 1912.

JOHNSTON, Mary, The Long Roll, Oct., 1911.

KANAVEL, Allen B., Infections of the Hand, page 473, Mar., 1912.
Kropf, S. A., Tuberculosis as a Disease of the Masses and How to Combat it, Aug., 1911.
Kelly, Howard A., Cyclopedia of American Medical Biography, page 713, July, 1912.
Kurella-Paul, Ceaser Lombroso, A Modern Man of Science, Nov., 1911.
Klopstock-Kowarsky, Manual of Clinical Chemistry, Microscopy and Bacteriology, page 655, June, 1912.

LARKIN, Edgar Lucien, Within the Mind Maze, of Mentonomy, the Law of the Mind, page 361, Jan., 1912.
Lea-Febiger, Progressive Medicine, page 362, Jan., 1912, Aug., 1911.
Larned, J. N., Life and Work of Wm. P. Letchworth, page, 592, May, 1912.

Lippincott, J. B. & Co., International Clinics, page 363, Jan., 1912.
Lord, Clara S., Receipts, Dec., 1911.
Lyle, H. Willoughly, Manual of Physiology, page 302, Dec., 1911.

MC'COMBS, Robert S., Diseases of Children for Nurses, page 416, Feb., 1912.
McConnell, Guthrie, Manuel of Pathology, page 595, May, 1912.
Martin, J. M., Practical Electro-Therapeutica and X-Ray Therapy, page 472, Mar., 1912.
May, Chas. H., Manual of the Diseases of the Eye, Oct., 1911.
Manuel of the International List of Causes of Death, page 474, Mar., 1912.
Mitchell, Clifford, Modern Urinology, page 596, May, 1912.
Morrow, Albert, Immediate Care of the Injured, page 656, June, 1912.
Montgomery, E. E., Practical Gynecology, 533, April, 1912.
Mortimer, J. D., Anaesthesia and Analgesia, page 414, Feb., 1912.
Motter-Wilbert, Digest of Comments on the Pharmacopoeia of the U. S. A. and on the National Formulary, page 535, April, 1912.
Mumford, James C., One Surgical Problems, Oct., 1911.
Munro, Henry S., Handbook of Suggestive Therapeutics, Applied Hypnotism and Psychic Science, Oct., 1911.
Musser-Kelly, Hand Book of Practical Treatment, page 598, May, 1912.

NEW and Non-Official Remedies-1912, page 656, June, 1912.

OERTEL, Horse, The Anatomic Histological Process of Bright's Disease and Their Relation to the Functional Changes, page 418, Feb., 1912.
Olser, Sir William, Practice of Medicine, page 475, March, 1912.

PATHOLOGIC Department of the Department of Clinical Psychiatry, Central Indiana Hospital for Insane, Report of the, page 712, July, 1912.
Patrick-Bassoe, Practical Medicine Series, page 475, March, 1912.
Page, Charles, Care of the Insane and Hospital Management, page 635, June, 1912.
Pels-Leuden, Prof., Surgical Operations, page 594, May, 1912.
Philips, Wendell Christopher, Diseases of the Ear, Nose and Throat, page 413, Feb., 1912.
Pilcher, Paul M., Practical Cystossopy and the Diagnosis of Surgical Diseases of the Kidneys and Urinary Bladder, Sept., 1911.
Pritchard, E. R., Health Hints, Aug., 1911.
Public Health and Marine Hospital Service of the U. S. for 1911, Annual Report of the Surgeon-General, page 598, May, 1912.
Potts, Charles S., Electricity, Medical and Surgical, Dec., 1911.
Preiser, George, Static Joint Diseases, page 475, Mar., 1912.

REESE, John J., Medical Jurisprudence and Toxicology, page 473, Mar., 1912.
Roberts, John B., Surgery of Deformities of the Face, page 661, June, 1912.
Roemer-Yoster, Text Book of Ophthalmology, page 658, June., 1912.
Ross, H. C., Further Researches into Induced Cell-Reproduction and Cancer, page 415, Feb., 1912.
Rurah, John, Manual of the Diseases of Infants and Children, Aug., 1911.

SANDERS, Georgiana, Modern Methods in Nursing, page 661, June, 1912.
Schudder, Charles L., Treatment of Fractures, page 357, Jan., 1912.
Scott, Kenneth, Refraction and Visual Acuity, page 360, Jan., 1912.
Schamberg, Jay F., Diseases of the Skin and the Eruptive Fevers, page 532, April, 1912.
Severance, Frank H., Studies of the Niagara Frontier, page 532, April, 1912.
Shumway, Frank, 38th Annual Report of the Secretary of the State Board of Health, page 596, May, 1912.
Simons, Charles E., A Manual of Clinical Diagnosis, Nov., 1911.
Snow, William Benham, Currents of High Potential and other Frequencies, page 299, Dec., 1911.
Still, George Frederic, Common Disorders and Diseases of Childhood, page 713, July, 1912

TAYLOR, E. W., Case Histories in Neurology, page 302, Dec., 1911.
Taylor, J. J., The Taylor Pocket Case Record, page 534, April, 1912.
Thornington, James, Retinoscopy, page 475, March, 1912.
Thornton, E. Quin, Manual of Materia Medica for Medical Students, Oct., 1911.
Todd, James C., Clinical Diagnosis, page 595, May, 1912.
Treves, Sir Frederick, Surgical Applied Anatomy, Dec., 1911.
Tweddell, Francis, A Mother's Guide, page 301, Dec., 1911.

UNDERHILL, Eugene, History of the Movement Registration of Nurses, page 473, Mar., 1912.

WALLACE, J. Sim, Prevention of Dental Caries, page 655, June, 1912.
Walker, Norman, An Introduction to Dermatology, page 416, Feb., 1912.
Ware, Martin W., Plaster of Paris and How to Use it, Aug., 1911.
Waugh, W. F., A Text Book of Alkaloidal Therapeutica page 416, Feb., 1912.

ZAHORSKY, John, Golden Rules of Pediatrics, Aug., 1911.

### GENERAL INDEX

ABORTIONISTS, Crusade against, edit., page 225, Nov., 1911.
Acute Pyelitis in Infancy, Edith R. Hatch, page 663, July, 1912.
Alcoholics, Buffalo Hospital for, Geo. H. McMichael, page 278, Dec., 1911.
Amputations, Traumatic Finger, A. H. Noehren, page 605, June, 1912.
Anterior Poliomyelitis, Fixation of Joints, Bernard Bartow and Wm. Ward Plummer, page 310, Jan., 1912.
Antivaccinationists, edit., page 168, Oct., 1911.
Asiatic Cholera, edit., page 48, Aug., 1911.
Athenian Epidemic of Meningitis, 1911, C. M. Mercurius, translation, page 452, March, 1912.
Austin Flood, E. H. Ashcraft, page 196, Nov., 1911.

BUFFALO Hospital for Alcoholics, George H. McMichael, page, 278, Dec., 1911.
Buffalo Water Supply, edit., page 345, Jan., 1912; page 519, April, 1912.

CALORIES and Fires, edit., page 409, Feb., 1912.
Carcinomata of Gastro-intestinal Tract, Wm. J. Mayo, page 305, Jan., 1912.
Carriers, Typhoid, edit., page 643, June, 1912.
Censorship, Dutchess County Resolution, edit., page 404, Feb., 1912.
Cholera Asiatica, edit., page 47, Aug., 1912.
Colds, Causes and Treatment, Sargent F. Snow, page 1, Aug., 1911.
College Life, Facts Concerning Physical Condition of Women During, Esther E. Parker, page 666, July, 1912.
Commission Business, page 224, Nov., 1911.
Compulsory Vaccination, edit., page 43, Aug., 1911.
Contract System, Evils of, Committee Report, page 82, Sept., 1911.
Contracted Pelves, Labor in Moderately, F. C. Goldsborough, page 126, Oct., 1911.
Conservative Ophthalmology, F. Parke Lewis, page 61, Sept., 1911.
Control Experiment and Enthusiasts, edit., page 99, Sept., 1911.
Corning Typhoid Epidemic, Frank S. Swain, page 622, June, 1912; edit., page 648, June, 1912.
Cotton or Wool, edit., page 227, Nov., 1911.
Criminal Intent, Question of, edit., page 101, Sept., 1911.
Cystitis, Silver Iodide in, James A. Gardner, page 321, Jan., 1912.

DENSITY of Population, edit., page 577, May, 1912.
Denutrition, Migraine, Esophoria, Fading Image, etc., in Eye Strain, George M. Gould, page 324, Jan., 1912.
Duodenal Ulcer, Perforation in, Edgar R. McGuire, page 613, June, 1912.
Dutchess County Resolutions Concerning State Censorship, edit., page 404, Feb., 1912.
Duties of Physicians in Regard to Insanity, Francis E. Fronczak, page 601, June, 1912.
Dysmenorrhoea, S. Y. Howell, page 543, May, 1912.

EDUCATION, Medica, Quality not Quantity, edit., page 103, Sept., 1911.

Education, Popular, edit., page 44, Aug., 1911.
Erie County, Report of Committee on Public Health of Medical Society of, page 386, Feb., 1912.
Esophoria, etc., in Eye Strain, George M. Gould, page 324, Jan., 1912.
Eye Strain, George M. Gould, page 324, Jan., 1912.
Evils of Contract Practice, Report of Committee, page 82, Sept., 1911.
Exchanges of 1845, page 580, May, 1912.
Expansion, Policy of, edit., page 41, Aug., 1911.

FACTS Concerning Physical Condition of Women during College Life, Esther E. Parker, page 666, July, 1912.
Fading Image, etc., in Eye Strain, George M. Gould, page 324, Jan., 1912.
Fees, Splitting of, edit., page 103, Sept., 1911.
Femur, Vicious Union of Shaft of, Edwin L. Bebee, page 253, Dec., 1911.
Finger Amputations, Traumatic, A. H. Noehren, page 605, June, 1912.
Fire-proof Schools, edit., page 523, Apr., 1912.
Fires and Calories, edit., page 409, Feb., 1912.
Fixation of Loose Joints, Bernard Bartow and Wm. Ward Plummer, page 310, Jan., 1912.
Future of Pharmacist, edit., page 284, Dec., 1911.

GASTRO-intestinal Tract, Carcinomata of, Wm. J. Mayo, page 305, Jan., 1912.
General Hospital, Buffalo, Bulletin of, page 155, Oct., 1911; page 340, Jan., 1912; page 514, Apr., 1912; July, 1912.
General Practitioner and Optometrist, edit., page 45, Aug., 1911.
Gonorrhoea, Treatment of Acute, Charles W. Bethune, page 121, Oct., 1911; Subacute, idem, page 185, Nov., 1911; Chronic, idem, page 256, Dec., 1911.
Graft, A Question of, edit., page 645, June, 1912.

HEART Murmurs, Timing of, Eli H. Long, page 375, Feb., 1912.
Heterophoria Superstition, George M. Gould, page 79, Sept., 1911.
Hypophysis, Normal and Pathologic Physiology of, Joseph L. Miller, page 365, Feb., 1912.

IMMIGRATION, Opposition to Restriction of, edit., page 646, June, 1912.
Inebriety, Some Statistic Studies of Cure of, T. D. Crothers, page 247, Dec., 1911.
Infancy, Acute Pyelitis in, Edith R. Hatch, page 663, July, 1912.
Inflamma tion of the Veru Montanum, and Posterior Urethra, with presentation of Instrument, James A. Gardner, page 609, June, 1912.
Insanity, Duties of Physicians, Francis E. Fronczak, page 601, June, 1912.
Instruction in Sexual Hygiene, edit., page 346, Jan., 1912.
Intra-articular Silk Ligaments, Bernard Bartow and Wm. Ward Plummer, page 310, Jan., 1912.
Intra-ligamentary Pregnancy and Retroperitoneal Tumors, John M. Lee, page 359, May, 1912.
Inventions, New, page 573, May, 1912; page 641, June, 1912; page 695, July, 1912.

JOINTS, Fixation of Loose, Bernard Bartow and Wm. Ward Plummer, Page 310, Jan., 1912.

LABOR in Moderately Contracted Pelves, Francis C. Goldsborough, page 126, Oct., 1911.
Labyrinthitis, Suppurative, Chester C. Cott, page 672, July, 1912.
Latent Sinusitis, George F. Cott, page 1, Aug., 1911.
Laurentian Waters, Problem of, edit., page 60, Oct., 1911.
Law as it Relates to Physicians and Buffalo Health Department, Francis E. Fronczak, page 477, April, 1912; page 563, May, 1912.
Lest We Forget, edit., page 461, Mar., 1912.
Ligaments, Intra-articular Silk, Bernard Bartow and Wm. Ward Plummer, page 310, Jan., 1912.
Location of Recent Graduates, edit., page 166, Oct., 1911.

MEDICAL Education, Quality not Quantity, edit., page 103, Sept., 1911.

Medical Expert Matter, New Departure in, S. W. Little, page 551, May, 1912.
Medicaments, Proposed List of Important, edit., page 222, Nov., 1911.
Meningitis, Floyd S. Crego page 421, March, 1912; Anthenian Epidemic of, 1911, C. M. Mercurius, translation, page 452, March, 1912; see Infantile Paralysis.
Migraine, etc., in Eye Strain, George M. Gould, page 324, Jan., 1912.
Murmurs, Timing of Heart, Eli H. Long, page 375, Feb., 1912.
Myoclonus, Associated with Unilateral Epileptoid Convulsions, J. W. Putnam and E. A. Sharpe, page 181, Nov., 1911.

NATIONAL White Cross Association, edit., page 406, Feb., 1912.
New and Non-official Remedies, Review, page 142, Oct., 1911; page 210, Nov., 1911; page 269, Dec., 1911; page 384, Feb., 1912.
New Departure in the Medical Expert Matter, S. W. Little, page 551, May, 1912.
New Inventions, page 573, May, 1912; page 641, June, 1912; page 695, July, 1912.
New Tri-state Directory, edit., page 349, Jan., 1912.
Niagara Frontier, Sanitation of Cities of, George E. Fell, page 486, Apr., 1912.
Normal and Pathologic Physiology of the Hypophysis, Joseph L. Miller, page 365, Feb., 1912.

OPHTHALMOLOGY, Conservative, E. Parke Lewis, page 61, Sept., 1911.
Opposition to Restriction of Immigration, edit., page 646, June, 1912.
Optometrist and General Practitioner, edit., page 45, Aug., 1911.
Orbit, Penetrating Wound of, with Dislodgement of Vomer, H. M. Cowper, page 336, Jan., 1912.
Otitis Media, Review of 100 Radicals, George F. Cott 243, Dec., 1911.

PARALYSIS, Infantile, Abstract, July, 1912.
Paralysis, Residual of Anterior Poliomyelitis, Bernard Bartow and Wm. Ward Plummer, page 310, Jan., 1912.
Penetrating Wound of Orbit with Dislodgement of Vomer, H. M. Cowper, page 336, Jan., 1912.
Perforation in Duodenal Ulcer, Edgar R. McGuire, page 613, J[illegible], 1912.
Pharmacist, Future of, edit., page 284, Dec., 1911.
Physical Condition of Women During College Life, Esther E. Parker, page 666, July, 1912.
Physician as Business Man., edit., page 164, Oct., 1911.
Polarimetry as Applied to Urinalysis, H. F. Lichtenberg, page 190, Nov., 1911.
Policewomen, edit., page 712, July, 1912.
Policy of Expansion edit., page 41, Aug., 1911.
Policy of Buffalo Medical Journal, edit., page 105, Sept., 1911.
Population, Density of, edit., page 577, May, 1912.
Postal Rates, edit., page 407, Feb., 1912.
Posterior Urethra and Veru Montanum, Inflammation, James A. Gardner, page 609, June, 1912.
Pregnancy, Intra-ligamentary and Retro-peritoneal Tumors, John M. Lee, page 539, May, 1912.
Prescriptions, Refilling, edit., page 226, Nov., 1911.
Problem of Laurentian Waters, edit., page 160, Oct., 1911.
Professional Ethics, edit., page 649, June, 1912.
Public Comfort Stations, edit., page 407, Feb., 1912.
Pyelitis, Acute in Infancy, Edith R. Hatch, page 663, July, 1912.

QUALITY, not Quantity in Medical Education, edit., page 103, Sept., 1911.

RECENT Graduates, Location of, edit., page 166, Oct., 1911.
Report of Cases Treated with Gonococcus Vaccine, John G. W. Knoll, page 550, May, 1912.
Residual Paralysis of Anterior Poliomyelitis, Bernard Bartow and Wm. Ward Plummer, 310, Jan., 1912.
Restriction of Immigration, edit., page 646, June, 1912.
Restriction of Immigration, edit., page 646, June, 1912.
Results from Use of Dowd's Phosphatometer and Tonic, Chauncey P. Smith, page 497, Apr., 1912.
Retroperitoneal Tumors and Intraligamentary Pregnancy, John M. Lee, page 539, May, 1912.

R-view of 100 Radicals, George F. Cott, page 243, Dec., 1911.
Rochester Public Health Association, E. G. Whipple, page 509, Apr., 1912.

SANE Christmas, edit., page 287, Dec., 1911.
Sanitation of Cities of Niagara Frontier, George E. Fell, page 486, Apr., 1912.
Segregation of the Tuberculous, edit., page 703, July, 1912.
Serum Therapy, W. S. Garlock, page 556, May, 1912.
Sexual Hygiene, Instruction in, edit., page 346, Jan., 1912.
Silk Ligaments, Intra-articular, Bernard Bartow and Wm. Ward Plummer, page 310, Jan., 1912.
Sinusitis, Latent, George F. Cott, page 1, Aug., 1911.
Some Statistic Studies of the Cure of Inebriety, T. D. Crothers, page 247, Dec., 1911.
Splitting of Fees, edit., page 103, Sept., 1911.
Suppurative Labyrinthitis, Chester C. Cott, page 672, July, 1912.
Syphilis, Treatment of, W. W. Quinton, page 14, Aug., 1911.

TECHNIC, John G. W. Knoll, page 446, March, 1912.
Thorax, Unusual Case of Inqury of, George Foy, page 604, June, 1912.
Three Years with Army of the Potomac, W. W. Potter, pages 18, 69, 132, 199, 261, 326, 377, 436, 500, 552, 625, 678, Aug., 1911—July, 1912.
Timing of Heart Murmurs, Eli H. Long, page 375, Feb., 1912.
Titanic Disaster, edit., page 575, May, 1912.
Traumatic Finger Amputations, A. H. Noehren, page 605, June, 1912.
Tri-state Directory, edit., page 349, Jan., 1912.
Tuberculosis Pavilian, Utica, edit., page 281, Dec., 1911.
Tuberculous, Segregation of, edit., page 703, July, 1912.
Typhoid Carriers, edit., page 643, June, 1912.
Typhoid Epidemic in Corning, Frank S. Swain, page 622, June, 1912; edit., 648, June, 1912.
Typhoid Mortality, What May Be Done., edit., page 405, Feb., 1912.

ULCER, Perforation in Duodenal, Edgar R. McGuire, page 613, June, 1912.
Unusual Case of Injury of Thorax, George Foy, page 604, June, 1912.
Urinalysis, Polarimetry as Applied to, H. F. Lichtenberg, page 109, Nov., 1911.
Use of Intra-articular Silk Ligaments, for Fixation of Loose Joints in Residual Paralysis of Anterior Poliomyelitis, Bernard Bartow and Wm. Ward Plummer, page 310, Jan., 1912.
Use of Silver Iodide in Urethritis and Cystitis, James A. Gardner, page 321, Jan., 1912.
Utica Tuberculosis Pavilion, edit., page 281, Dec., 1911.

VACCINATION, Compulsory, edit., page 43, Aug., 1911.
Vaccination, Antivaccinationists, edit., page 168, Oct., 1911.
Vaccine, Gonococcus, John G. W. Knoll, page 550, May, 1912.
Veru Montanum and Posterior Urethra, Inflammation, James A. Gardner, page 609, June, 1912.
Vicious Union of Shaft of Femur, Edwin L. Bebee, page 253, Dec., 1911.
Vincent's Angina, V. M. Griswold, page 431, March, 1912.
Vomer, Penetrating Wound of Orbit with Dislodgement of., H. M. Cowper, page 336, Jan., 1912.

WATER Drinking and Drinking water, Abstract, Kuhner, page 635, June, 1912.
Water Supply, Buffalo, edit., page 345, Jan., 1912; page 519, April, 1912; page 574, May, 1912.
Weather Bureau, Suggestion for, edit., page 348, Jan., 1912.
Wool or Cotton, edit., page 227, Nov., 1912.

Obstetrical Charts in Colors.—Ten full plates 12x9 illustrating and briefly describing the following obstetrical positions.

1. Diameters of foetal head, pelvic brim and planes of pelvis.
2. Head presentations.
3. Mechanism in vertex presentations.
4. Mechanism in left occipito-anterior presentation.
5. Face presentations.
6. Mechanism in face presentations.
7. Right mento-posterior position.
8. Breech presentations.
9. Mechanism in Breech presentations.
10. Transverse positions.

These plates will be sent in book form to any address on receipt of 25 cents, postpaid. Battle & Co., Saint Louis( Missouri.

---

Were it not that the use of opium, morphine, heroin, and codeine is so frequently attended by distress and even danger, there couud be no argument to be advanced in favor of Papine; but when it may be said that the greatest of Papine's faults is not so weighty as the least fault of opium and morphine, one does not wonder that the use of Papine is growing wider and wider.

Papine's field of usefulness is not bounded by its power to soothe pain. Its sedative influence on inflammed mucosae point to it as a most desirable constituen of mixtures serviceable in bronchial and pulmonary inflammations.

In short, it may be said of Papine's uses that whatever is expected of opium may be likewise expected of Papine.

---

"Both in my practice in New York City and particularly during the summer when I am located at Richfield Springs, N. Y., a resort where thousands of rheumatic and gouty patients take the Sulphur Baths, I have prescribed Tongaline extensively and it has always proved most satisfactory."

"I would state that owing to the care and skill used in its manufacture, as also because it is always uniform and is well borne by the stomach, Tongaline stands foremost among the ready-made prescriptions for rheumatism, neuralgia, grippe, gout, etc. Besides the conscientious practitioner hesitates about having sucha complicated prescription as Tongaline prepared by a pharmacist,.because even if the latter had fresh and pure ingredients, he has not the facilities to compound them properly nor could he do so in any reasonable time."

